# The effect of the macrobiotic Ma-Pi 2 diet vs. the recommended diet in the management of type 2 diabetes: the randomized controlled MADIAB trial

**DOI:** 10.1186/1743-7075-11-39

**Published:** 2014-08-25

**Authors:** Andreea Soare, Yeganeh M Khazrai, Rossella Del Toro, Elena Roncella, Lucia Fontana, Sara Fallucca, Silvia Angeletti, Valeria Formisano, Francesca Capata, Vladimir Ruiz, Carmen Porrata, Edlira Skrami, Rosaria Gesuita, Silvia Manfrini, Francesco Fallucca, Mario Pianesi, Paolo Pozzilli

**Affiliations:** 1 Department of Endocrinology and Diabetes, University Campus Bio-Medico, Via Alvaro del Portillo 21, 00128 Rome, Italy; 2 Unit of Dietology and Diabetology, Sandro Pertini Hospital, Via dei Monti Tiburtini 385, 00157 Rome, Italy; 3 Department of Laboratory Medicine, University Campus Bio-Medico, Rome, Italy; 4 Department of Biochemistry and Physiology, Institute of Nutrition and Food Hygiene, Infanta 1158, 10300 Havana, Cuba; 5 Clinical Assay Direction, Finlay Institute, Avenue 27, No. 19805, La Coronela, La Lisa 11600, Havana, Cuba; 6 Center of Epidemiology, Biostatistics and Medical Information Technology, Polytechnic Marche University, Via Tronto 10A, 60020 Ancona, Italy; 7 Department of Clinical Sciences, La Sapienza University II Faculty, Via di Grottarossa 1035/1039, 00189 Rome, Italy; 8 International Study Center for Environment, Agriculture, Food, Health and Economics, Via San Nicola, 62029 Rome, Italy

**Keywords:** Fasting blood glucose, Macrobiotic diet, Type 2 diabetes

## Abstract

**Background:**

Diet is an important component of type 2 diabetes therapy. Low adherence to current therapeutic diets points out to the need for alternative dietary approaches. This study evaluated the effect of a different dietary approach, the macrobiotic Ma-Pi 2 diet, and compared it with standard diets recommended for patients with type 2 diabetes.

**Methods:**

A randomized, controlled, open-label, 21-day trial was undertaken in patients with type 2 diabetes comparing the Ma-Pi 2 diet with standard (control) diet recommended by professional societies for treatment of type 2 diabetes. Changes in fasting blood glucose (FBG) and post-prandial blood glucose (PPBG) were primary outcomes. HbA_1c_, insulin resistance (IR), lipid panel and anthropometrics were secondary outcomes.

**Results:**

After correcting for age, gender, BMI at baseline, and physical activity, there was a significantly greater reduction in the primary outcomes FBG (95% CI: 1.79; 13.46) and PPBG (95% CI: 5.39; 31.44) in those patients receiving the Ma-Pi 2 diet compared with those receiving the control diet. Statistically significantly greater reductions in the secondary outcomes, HbA_1c_ (95% CI: 1.28; 5.46), insulin resistance, total cholesterol, LDL cholesterol and LDL/HDL ratio, BMI, body weight, waist and hip circumference were also found in the Ma-Pi 2 diet group compared with the control diet group. The latter group had a significantly greater reduction of triglycerides compared with the Ma-Pi 2 diet group.

**Conclusions:**

Intervention with a short-term Ma-Pi 2 diet resulted in significantly greater improvements in metabolic control in patients with type 2 diabetes compared with intervention with standard diets recommended for these patients.

**Trial registration:**

Current Controlled Trials ISRCTN10467793.

## Background

Type 2 diabetes is currently one of the most challenging problems facing national healthcare systems worldwide [1]. Nutritional therapy (as part of life-style intervention), with or without additional drug treatment, represents an effective option for managing this disease [2]. Formulating a universal diet for type 2 diabetes patients can be difficult, since cultural preferences and economic conditions influence patient acceptance of, and adherence to, recommended diets [3].

In general, consumption of healthy, plant-based diets which are low in saturated fat and refined carbohydrates but high in whole grains, vegetables, legumes and fruits, coupled with appropriate exercise regimens, is recommended for patients with type 2 diabetes [4-7]. Additionally, reduction in the intake of carbohydrates with a high glycemic index has been shown to result in significant improvements in glucose tolerance and body weight when compared with conventional low-fat diets with similar energy content [8]. The International Diabetes Federation (IDF) and the American Diabetes Association (ADA) have issued diet recommendations for patients with type 2 diabetes [9,10], however, alternative approaches need to be investigated because of the frequent low adherence of the currently recommended diets in the management of type 2 diabetes [11].

Macrobiotic diets, originally derived from an ancient Eastern philosophy of life, and updated for Western culture by the Japanese philosopher Georges Ohsawa [12], contain a large proportion of whole grains. The Ma-Pi 2 diet, conceived by Mario Pianesi, is a kind of macrobiotic diet; it is high in dietary fiber, which is in line with dietary recommendations by the Academy of Nutrition and Dietetics [13]. High-fiber diet may induced several health benefits such as prevention or reduction of bowel disorders and decreased risk of the development of coronary heart disease and type 2 diabetes [14,15]. The Ma-Pi 2 diet is also rich in complex carbohydrates, whole grains, vegetables and legumes, fermented products, sea salt and green tea, without fat or protein from animal sources (including milk and dairy products) and no added sugars. These features are designed to achieve optimal glucose control, lower insulin requirement, prolong the time of glucose absorption, increase insulin sensitivity, reduce total cholesterol and triglyceride levels in plasma, improve body weight control and lower systemic blood pressure [16-19]. Additionally, the Ma-Pi 2 diet appears to have antioxidant properties and prebiotic or probiotic effects [20]. This may alter the composition of gut microbiota, which in turn may affect the glycemic control [21,22].

In previous uncontrolled intervention studies of 3-weeks’ duration, patients with type 2 diabetes following the Ma-Pi 2 diet have exhibited reduced HbA_1c_, cholesterol, triglycerides, and blood pressure [23,24]. We report here on the first randomized comparative trial which compares the Ma-Pi 2 diet with the dietary guidelines for type 2 diabetes recommended by professional societies in Italy [25].

## Methods

### Trial design

The study was a 21-day, controlled open-label trial in which participants were randomized (1:1 ratio) to the Ma-Pi 2 macrobiotic diet or to a diet based on dietary recommendations guidelines for type 2 diabetes [25]. The trial took place in the spring of 2013 at 2 closed site hotels in Italy. Throughout the trial, participants stayed at two different hotels according to the type of diet they were randomized to. The hotels were localized in the same geographic area, very close to each other, approximately a 20 minutes’ drive distance between them. Patients were recruited from the Endocrinology and Diabetes Department at University Campus Bio-Medico. The trial was conducted in accordance with the Declaration of Helsinki and the Good Clinical Practice guidelines and approved by the Institutional Review Board of University Campus Bio-Medico.

### Inclusion and exclusion criteria

Overweight or obese (BMI 27–45 kg/m^2^) males and females, aged 40–75 years affected by type 2 diabetes were recruited by the medical team during regular visits to the Department of Endocrinology and Diabetes of the University Campus Bio-Medico in Rome, Italy. Additional inclusion criteria were a diagnosis of type 2 diabetes at least 1 year prior to the start of the trial and management with dietary intervention, oral hypoglycemic drugs (OADs), or both for 6 months prior to study entry. Identification of the presence of associated metabolic syndrome was made in accordance to the National Cholesterol Education Program Adult Treatment Panel III criteria, even though it was not an inclusion criteria [26]. Exclusion criteria included current use of insulin, or use at any time in the 2 years prior to the study, current use of corticosteroid therapy or any other drug that could interfere with carbohydrate metabolism, alcohol abuse, pregnancy, or already following a macrobiotic diet.

### Interventions

Participants’ eating habits prior to study entry were assessed using qualitative and quantitative questionnaires, and the energy and nutritional content of their diet was evaluated based on the answers given. Group assignment to treatment was blinded for personnel involved in collection of follow-up data and for personnel analyzing blood samples until data was locked for statistical analysis.

Participants were randomized to either the Ma-Pi 2 diet or a control diet according to the dietary guidelines for type 2 diabetes. The Ma-Pi 2 diet consisted of whole grains, vegetables and legumes. Beicha tea (roasted green tea) represented the main source of liquids, while the control diet was adapted to the Mediterranean culinary style. For both groups, energy intake was restricted by limiting calories to 1900 kcal/day and 1700 kcal/day for males and females, respectively. The diets were isocaloric but differed in nutrient composition. Ma-Pi 2 diet derived 72% of energy from carbohydrate, 18% from fat, and 10% energy from protein, fiber equal to 30 g/1000 kcal, while the control diet 50% from carbohydrate, 20% from protein, and 30% from fat, fiber ≥20 g/1000 kcal. Alcohol consumption was forbidden. Both diets provided 5 meals per day, with energy intake being divided between meals, 20% calories at breakfast, 30% calories at lunch and 30% calories at dinner. Two snacks were administered at approximately 2.5 hours after breakfast and lunch, respectively, each contributing 10% of the calories per day (Additional file 1).

A 10-days menu cycle was devised for both diets and then repeated at the end of the period for another 11 days. Each daily menu was carefully planned and completed with recipes for each dish. Nutritional analysis and menu planning was developed with MètaDieta® Software using the Italian Food Composition Tables edited by the National Institute for Food and Nutrition Research (INRAN) [27]. Each menu cycle was ready a week before the beginning of the study to ensure that each recipe was tested by the cooks in order to reproduce it consistently. At this stage and throughout the intervention study, dietitians checked and weighed each portion size before and after cooking to make sure that subjects in both groups consumed equivalent amounts of energy and that macronutrient content was respected. Each subject was informed that a plate waste greater than or equal to 5% of the total daily food amount meant dismissal from the trial. Throughout the trial, participants stayed at two different hotels according to the type of the diet they were randomized. Meal consumption was strictly controlled in order to evaluate dietary compliance during the trial. Participants had their meals together with physicians and dietitians who could check their adherence to study protocol. Dietary adherence was defined as absence of any transgression from the assigned diet. Those subjects who attended meal session for fewer than 20 out of 21 days were considered non-adherent.

Participants were asked not to alter their exercise habits during the intervention period. Physical activity was assessed daily using a pedometer (Tri-axial activity monitor XL-18/XL-18 CN-AND A&D Medical-California-USA). In addition they were instructed to continue their pre-study OAD doses without modification throughout the study, unless hypoglycemic symptoms were accompanied by a capillary glucose reading <70 mg/dL. In such cases hypoglycemic medications were reduced for participant safety.

### Outcome measures

The primary outcomes of this study were the percentage change in Fasting Blood Glucose (FBG) and Post Prandial Blood Glucose (PPBG) levels from baseline (T0) to the 21^st^ day of treatment (T21) in Ma-Pi 2 group compared to the control group. Secondary outcomes included percentage change from baseline in plasma concentrations of HbA_1c_, total cholesterol, LDL cholesterol (LDLc), HDL cholesterol (HDLc), LDL/HDL ratio, and percentage change from baseline of insulin resistance, body weight, BMI, waist and hip circumference, and number of patients who achieved target values of FBG ≤110 mg/dl and PPBG ≤140 mg/dl.

FBG and PPBG were measured daily from T0 to T21 by the medical staff using capillary blood glucose meters (Bluecare, Biochemical Systems International, Arezzo, Italy). The fasting blood glucose was measured right before meals and the post-prandial blood glucose was measured 2-h after lunch. For all participants, venous blood samples were obtained early in the morning after a 12 hour fasting period. All biochemical and anthropometrical measures were assessed at T0 and T21 by the central laboratory (University Campus Bio-Medico, Rome). Insulin resistance was calculated using the homeostasis model assessment of insulin resistance (HOMA-IR) [28].

Body weight was measured at T0 and T21 before breakfast using a digital scale accurate to 0.1 kg, waist circumference was measured with a tape measure placed 2.5 cm above the umbilicus. Hip circumference was measured at the maximal protrusion of the buttocks.

All patient and investigator-reported adverse events (AEs) were recorded at each visit.

### Statistical analyses

This study required 28 randomized patients per group with 80% power to detect a difference of at least 12 percentage points reduction in mean FBG and PPBG from baseline between Ma-Pi 2 and control groups (with a 2-sided type I error at 0.05), assuming a standard deviation of ≤15% and a maximum dropout rate of 11%.

To detect a 0.25 percentage point between-group difference in HbA_1c_ from baseline (the main secondary endpoint) with 80% power, a p value at the 5% level with an assumed SD of 0.3% and 10% estimated withdrawal, 27 participants were required per group.

The primary analysis was based on a modified intention-to-treat principle and it was carried out for all patients who had FBG and PPBG results for at least the first week following randomization. A non-parametric approach was chosen for the statistical analysis since outcome variables were found to be of non-normal distribution (using the Shapiro test). Quantitative variables were summarized using percentiles (median and interquartile range). Comparisons between treatment groups were performed using the Wilcoxon rank-sum test and 95% confidence intervals (95% CI) for median values. Absolute and percentage frequencies were used for qualitative variables and the Fisher exact test was applied for group comparisons.

The 2 groups were compared at T0 to test whether patients were similar in terms of demographic and anthropometrics characteristics, and lipid and carbohydrate metabolic parameters. The percent variations between values measured at T0 and T21 were calculated as efficacy variables for the primary (FBG and PPBG), and secondary end-points (HbA_1c_, HOMA-IR, total cholesterol, LDLc, HDLc, LDL/HDL ratio, triglycerides, BMI, waist and hip circumference).

**Figure 1:**
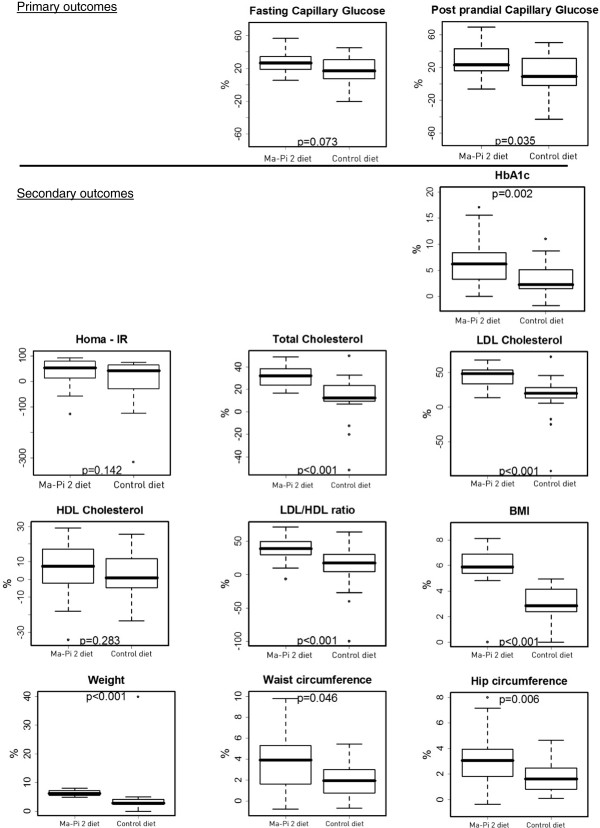
**Reduction in primary and secondary outcomes from baseline to T21 in the Ma-Pi 2 group vs. controls.** Primary outcomes - Percentage change in FBG and PPBG from baseline to T21 in the Ma-Pi 2 group vs. control group. The figure represents the results of the bivariate analysis when the Ma-Pi 2 and control groups were compared in the percentage change between T0 and T21 of the efficacy variables (non-adjusted values). Secondary outcomes - Percentage change in HbA_1c_, lipid panel, BMI, weight, hip circumference and waist circumference from baseline to T21 in the Ma-Pi 2 group vs. control group. The figure represents the results of the bivariate analysis when the Ma-Pi 2 and control groups were compared in the percentage change between T0 and T21 of the efficacy variables (non-adjusted values).

A bivariate analysis was performed to compare measures changes between the Ma-Pi 2 diet group and the control group and results were graphically represented by means of box-plots (Figure 1). A linear quantile regression analysis [29] was performed to estimate the effect of the diet (Ma-Pi 2 vs. control, explanatory variable) on the median percentage change occurred in each measured variable as (dependent variables), between T0 and T21. Each model was adjusted for those variables that could potentially affected the percent changes in the dependent variables, i.e. gender, age, BMI at baseline, and physical activity done by subjects during 21 days of the treatment (measured as the median number of kilometers walked per day). When HOMA-IR was analyzed, the wrist circumference was included in the model as covariate for adjustment. Wrist circumference is recognized a valid and easy marker to measure insulin resistance [30,31]. The results of linear quantile regression analysis were expressed as point and interval estimates of regression coefficients; when the coefficient was positive, the measured variable reduction was related to Ma-Pi 2 diet effect and when the coefficient was negative the reduction was related to the control diet. When the 95% CI’s did not include zero, regression coefficients were considered statistically significant (Table 1).

**Table 1:** Effect of Ma-Pi 2 vs. control diet on primary and secondary outcomes

**Dependent variables (% changes)**	**Regression coefficients**	95% CI**
Fasting Blood Glucose (mg/dl)	6.82	1.79; 13.46
Postprandial Blood Glucose (mg/dl)	11.48	5.39; 31.44
HbA_1c_ (%)	3.46	1.28; 5.46
HbA_1c_ (mmol/mol)	3,08	1,21, 5,13
HOMA-IR*	15.14	3.65; 37.51
Total Cholesterol (mg/dl)	18.61	11.52; 22.63
LDL Cholesterol (mg/dl)	26.40	21.37; 34.50
HDL Cholesterol (mg/dl)	4.82	−5.69; 10.22
LDL/HDL Ratio	31.54	20.63; 36.52
Triglycerides (mg/dl)	−11.04	−24.41; −2.92
BMI (kg/m2)	2.99	2.42; 3.59
Weight (kg)	3.04	2.39; 3.58
Waist circumference (cm)	2.01	0.08; 3.17
Hip circumference (cm)	1.22	0.4; 1.94

*Wrist circumference was included as covariate for HOMA-IR.

**Coefficients are statistically significant when the 95% CI’s does not include zero.

Legend: Results of multiple quantile regression analysis evaluating the effect of Ma-Pi 2 diet on the percentage changes (T0-T21) in biochemical and anthropometrical measures using a multiple quantile regression model (adjusted value for age, gender, physical activity and BMI at baseline).

**Figure 2:**
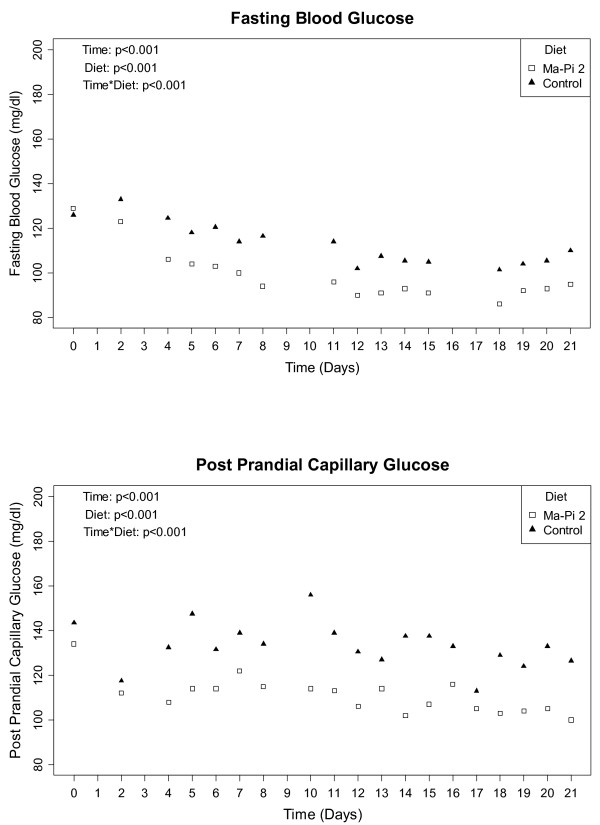
**Linear distribution of the daily blood glucose values during the 21 days in and between groups.** The figure represents the results of a non-parametric longitudinal analysis of all daily FBG and PPBG values between baseline and T21 in the Ma-Pi 2 group and the control group.

Patients with values of FBG ≤110 mg/dl and of PPBG ≤140 mg/dl were considered to have reached target values. Group comparisons in terms of percentage of patients achieving and maintaining those target levels after 21 days were performed using McNemar’s test to estimate the differences between proportions in the 2 groups and their relative 95% CI’s; when the 95% CI’s did not include zero, the differences were statistically significant (Table 2).A ranked-based nonparametric analysis for longitudinal data was used to compare the time trend in absolute values of FBG and PPBG over 21 days of dietary intervention in Ma-Pi 2 and control groups. The effect of diet, time and their interaction were evaluated; results were graphically represented by plotting the median values of the daily measurements of FBG and PPBG in the two groups (Figure 2).

**Table 2:** Number of patients who achieved target blood glucose levels after 21 days of dietary treatment

	**Ma-Pi 2 diet (n. 25)**	**Control diet (n. 26)**		95% CI*	
**Fasting blood glucose (target value ≤ 110 mg/dl)**	**n (%)**	**n (%)**	**Difference (%)**	**LL**	**UL**
Target achievement**	19 (100)	9 (45)	55.00	33.20	76.80
Target maintenance**	6 (100)	5 (83.33)	16.67	−13.15	46.49
**Post-prandial blood glucose (target value ≤ 140 mg/dl)**					
Target achievement	11 (100)	8 (53.33)	46.67	21.42	71.91
Target maintenance	14 (100)	9 (81.82)	18.18	−4.61	40.97

*McNemar test. Coefficients are statistically significant when the 95% CI’s does not include zero.

**The percentage of patients that achieved the glycemic target level (both fasting and 2-h after lunch capillary glucose) between T0 and T21 and the percentage of patients that maintained the glycemic target level (both fasting and 2-h after lunch capillary glucose) between T0 and T21, in the Ma-Pi 2 group compared with the control group.

All statistical analyses were performed using R statistical package (Foundation for Statistical Computing, Vienna, Austria) and statistical significance was assessed at a level of probability of 0.05.

## Results

A total of 200 patients were screened and 56 were considered eligible for recruitment.

**Figure 3:**
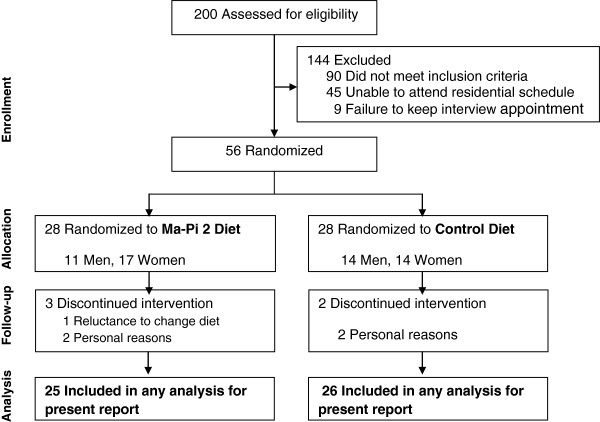
Consort diagram reflecting flow of study participants through the study.

Baseline demographic and clinical features of patients are presented in Table 3. There were no significant differences between groups. Analysis of diets in the 6 months prior to the trial showed little difference between groups, with an average daily energy intake of 1988 (SD 368.2) kcal for the Ma-Pi 2 group and 1964 (SD 294.3) kcal for the control group (p = 0.802). Nutritional composition was also similar in the Ma-Pi 2 group (18.2% protein, 36.2% fat, 45.6% carbohydrate, 10.3 g/1000 kcal of fiber) versus the control group (19.3% protein, 35.4% fat, 45.3% carbohydrate, 10.8 g/1000 kcal of fiber).The 56 eligible patients were randomly assigned to the Ma-Pi 2 diet group (n = 28) and the control group (n = 28). Reasons for exclusion were failure to meet inclusion criteria (n = 90), inability to attend residential schedule (n = 45), and failure to keep interview appointment (n = 9). The CONSORT diagram reflecting flow of study participants through the study is shown in Figure 3. Both groups experienced drop outs after randomization but before receiving any intervention; 3 patients in the Ma-Pi 2 diet group and 2 in the control group. Reasons for discontinuation included patient reluctance to change diet (1 patient in the Ma-Pi 2 group) and personal problems that prevented patients remaining in the assigned hotel for the study duration (2 patients in the Ma-Pi 2 group and 2 patients in the control group). Therefore a total of 51 patients, 25 in the Ma-Pi 2 group and 26 in the control group, completed the trial and were included in the modified ITT (mITT) analysis. The modified intention-to-treat was carried out for all patients who had FBG and PPBG results for at least the first week following randomization.

**Table 3:** Baseline characteristics according to study group

**Median [95% CI]**	**Ma-Pi 2 diet**	**Control diet**	p value*
	**(n = 25)**	**(n = 26)**	
Age (years)	67 [63.8; 70.2]	65 [62.2; 67.8]	0.213
Male, n (%)	11 (44)	14 (53,8)	0.580^#^
Duration of T2D (years)	7 [3.9; 10.01]	4.5 [1.1; 7.9]	0.494
On Metformin, n (%)	13 (61.9)	20 (90.9)	0.034^#^
On other OADs, n (%)	7 (33.3)	10 (45.5)	0.537^#^
Fasting blood glucose (mg/dl)	118 [104.7; 131.3]	127 [115.2; 138.8]	0.888
HbA_1c_ (%)	6.7 [6.2; 7.2]	6.8 [6.6; 7.0]	0.197
HbA_1c_ (mmol/mol)	50 [44; 55]	51 [49; 53]	0.228
HOMA-IR	3.1 [2.1; 4.0]	3.3 [2.1; 4.5]	0.910
Total cholesterol (mg/dl)	176 [156.4; 195.6]	180.5 [160.7; 200.3]	0.865
LDL cholesterol (mg/dl)	102 [86.5; 117.5]	101.5 [89.1; 113.9]	0.749
HDL cholesterol (mg/dl)	46 [41.6; 50.4]	48.5 [44.5; 52.5]	0.428
LDL/HDL ratio	2.4 [2.1; 2.7]	2 [1.7; 2.3]	0.243
Triglycerides (mg/dl)	114 [83.7; 144.3]	119 [97.6; 140.4]	0.644
BMI (kg/m^2^)	31.3 [29.1; 33.5]	32 [29.5; 34.5]	0.486
Weight (kg)	81.1 [73.7; 88.5]	87 [79.0; 94.9]	0.713
Waist circumference (cm)	107.6 [102.3; 112.9]	105.2 [99.8; 110.6]	0.963
Hip circumference (cm)	108.7 [102; 115.4]	109.7 [104.7; 114.7]	0.821

*Wilcoxon rank sum test.

^#^Fisher exact test.

Legend: All data showed are a median [95% Confidence Interval CI] unless otherwise noted. Coefficients are statistically significant when the 95% CI’s does not include zero.

Daily average energy intake during the trial was 1803 (SD 95.2) kcal (11.8% protein, 15.2% fat, and 73.0% carbohydrates, with 29 g/1000 kcal fiber) in the Ma-Pi 2 group, and 1798 (SD 106.3) kcal (18.4% protein, 32.3% fat, and 49.3% carbohydrates, with 20.5 g/1000 kcal fiber) for the control group (p = 0.860). For the Ma-Pi 2 diet the daily average amount of carbohydrate was 335.7 g. As for the control diet the daily average amount of carbohydrate was 235.8 g (p < 0.001).Figure 1 shows the primary and secondary outcomes results. A significant reduction was observed in both groups for FBG and for PPBG. The reduction in PPBG (p = 0.035) levels was significantly greater in patients in the Ma-Pi 2 group compared with those in the control group.

Regarding secondary outcomes, the Ma-Pi 2 group showed a significantly greater reduction in HbA_1c_ (p = 0.002) levels compared with those in the control group. Significantly greater median percentage reductions were observed for total cholesterol, LDLc, and the LDL/HDL ratio in the Ma-Pi 2 group versus the control group (p < 0.001). No significant change was observed for HDLc levels from baseline between groups and within groups (p = 0.283). Median BMI and weight were significantly reduced in both groups, and those reductions were significantly higher in the Ma-Pi 2 group compared with the control group (p < 0.001) (Table 4).A non-parametric longitudinal data analysis of the daily FBG and PPBG evidenced a trend curve reduction in both groups over time (p < 0.001), with a higher tendency in the macrobiotic Ma-Pi 2 diet group (p < 0.001). Furthermore, the duration and the type of diet positively influenced the reduction of both FBG and PPBG (p < 0.001) (Figure 2).

**Table 4:** Primary and secondary outcomes comparison between groups at baseline and after 21 days on the prescribed diets

**Median [1**^**st**^**-3**^**rd **^**quartiles]**	**Ma-Pi 2 diet (n = 25)**				**Control diet (n = 26)**				
	**Baseline**	**T21**	**Absolute change**	**Percent change**	**Baseline**	**T21**	**Absolute change**	**Percent change**	p value*
**Fasting blood glucose (mg/dl)**	**129 (111; 149)**	**95 (91; 100)**	**34 (21; 44)**	**26.2 (18.7; 34.1)**	**126 (112; 150)**	**110 (99; 115)**	**22.8 (8.1; 44.6)**	**16.9 (6.9; 30.1)**	**0.073**
**Post prandial blood glucose (mg/dl)**	**134 (112; 179)**	**100 (94; 106)**	**34 (18; 75.5)**	**23.1 (15.9; 42.9)**	**144 (128; 210)**	**127 (108; 151)**	**15.3 (−1.6; 58.6)**	**8.7 (−1.9; 50.3)**	**0.035**
**HbA**_**1c **_**(%)**	**6.7 (6.2; 7.7)**	**6.3 (5.9; 7)**	**0.4 (0.2; 0.6)**	**6.2 (3.3; 8.3)**	**6.8 (6.4; 7.1)**	**6.6 (6.2; 7)**	**0.2 (0.1; 0.4)**	**2.2 (1.5; 5)**	**0.002**
**HOMA-IR**	**3.1 (1.2; 4.1)**	**0.98 (0.61; 1.98)**	**1.3 (0.1; 2.9)**	**51.4 (11.7; 78.3)**	**3.3 (0.9; 4.6)**	**1.6 (1.2; 2.4)**	**1.4 (−0.2; 2.3)**	**40.4 (−21.9; 62)**	**0.142**
**Total cholesterol (mg/dl)**	**176 (155; 217)**	**120 (102; 138)**	**57 (40; 65)**	**32 (23.8; 38.6)**	**180.5 (150.2; 210.8)**	**154 (133; 185)**	**24 (16; 43)**	**12.3 (9.9; 23)**	**< 0.001**
**LDL cholesterol (mg/dl)**	**102 (84; 133)**	**62 (39; 69)**	**45 (33; 58)**	**47.9 (33.5; 53.6)**	**101.5 (84.8; 123.5)**	**81 (66; 105)**	**20 (12.3; 31.3)**	**19.7 (13.5; 27.8)**	**< 0.001**
**HDL cholesterol (mg/dl)**	**46 (38; 52)**	**42 (36; 51)**	**4 (−1; 8)**	**7.5 (−2.2; 17.1)**	**48.5 (43; 55.8)**	**47 (40; 56)**	**0.5 (−2; 6.8)**	**0.9 (−4.4; 11.5)**	**0.283**
**LDL/HDL ratio**	**2.4 (2; 3)**	**1.3 (0.9; 2.4)**	**0.9 (0.5; 1.3)**	**38.5 (29.3; 49.9)**	**2 (1.8; 2.6)**	**1.8 (1.3; 2.4)**	**0.4 (0.1; 0.7)**	**17.2 (5.9; 29.5)**	**< 0.001**
**BMI (kg/m**^**2**^**)**	**31.3 (29.9; 36.8)**	**29.5 (28.2; 34.6)**	**2 (1.8; 2.4)**	**5.9 (5.4; 6.9)**	**32 (28.2; 35.9)**	**30.8 (27.3; 34.6)**	**1 (0.7; 1.3)**	**2.9 (2.4; 4.1)**	**< 0.001**
**Weight (kg)**	**81.1 (75.6; 99.1)**	**77 (70; 94)**	**5.5 (4.4; 6)**	**6.3 (5.6; 7.2)**	**87 (79; 103.9)**	**83 (75; 100)**	**2.8 (2; 3.6)**	**2.9 (2.4; 4.2)**	**< 0.001**
**Waist circumference (cm)**	**107.6 (97.9; 114.7)**	**103 (96; 112)**	**4.2 (1.4; 6.5)**	**3.9 (1.6; 5.3)**	**105.2 (101.6; 117.5)**	**103 (101; 113)**	**2.3 (0.9; 4)**	**1.9 (0.9; 2.9)**	**0.046**
**Hip circumference (cm)**	**108.7 (102.5; 23.8)**	**105 (99; 120)**	**3.4 (2; 5.2)**	**3.1 (1.8; 4)**	**109.7 (102.6; 118.4)**	**108 (101; 118.4)**	**1.8 (1; 2.5)**	**1.6 (0.8; 2.4)**	**0.006**

Legend: *P-value by Wilcoxon rank-sum test are referred to percent changes between the two treatment groups.

The results of the multiple quantile regression analysis (adjusted for age, gender, BMI at baseline, and physical activity) are reported in Table 1. A significantly higher percentage reduction of FBG, PPBG, and HbA_1c_ were associated with the Ma-Pi 2 diet, and a statistically significant higher effect on the percentage reductions in the same group was obtained for total cholesterol, LDL-cholesterol and LDL/HDL ratio, BMI, weight, and waist and hip circumference compared with the control group. The Ma-Pi 2 diet group experienced a significantly greater reduction of HOMA-IR (adjusted for wrist circumference) compared with the control group. No statistically significant changes in HDL-cholesterol levels were noted in either group, whereas the control diet group showed a significantly higher effect in the reduction of triglyceride levels compared to the Ma-Pi 2 group even though a reduction in triglycerides levels was obtained in both groups, none of the study participants had triglyceride values in the higher range after intervention.

**Figure 4:**
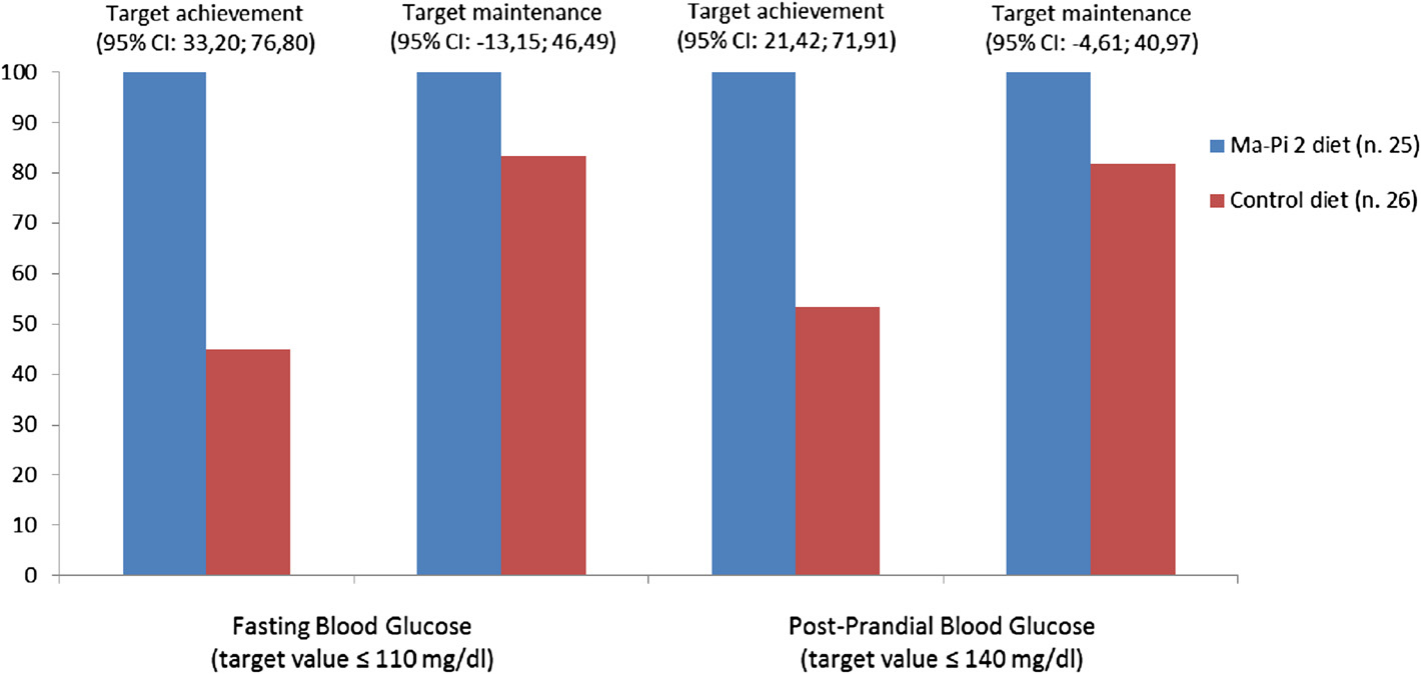
Percentage of patients who achieved or maintained target blood glucose levels after 21 days of dietary treatment.

Table 2 shows the results obtained by comparing both groups in the achievement and maintenance of FBG and PPBG. The percentage of patients who achieved FBG and PPBG target levels was significantly higher in the Ma-Pi 2 group compared with the control group. No significant difference was found in the percentage of patients that maintained the glycemic target level (both fasting and postprandial capillary glucose) between T0 and T21 (Figure 4). For some patients, OAD therapy had to be reduced to avoid hypoglycemia. A statistically significant reduction in OAD therapy occurred in the Ma-Pi 2 group compared with the control group (p = 0.018); from a total of 7 patients on sulfonylurea or glinide and/or DPP-4 inhibitor treatment at baseline, 5 suspended treatment (resulting in a total daily reduction of 8.5 mg of glinide, 200 mg glicazide, and 10 mg glibencamide). There were no episodes of hypoglycaemia in both the intervention group and the control group even though in the Ma-Pi 2 group there was a tendency for lower blood glucose levels. In the control group only 1 patient suspended OAD treatment (equating to a total reduction of 60 mg glicazide). Compliance was good in all patients, with no meals missed by any patient in either group. There were no reported severe AEs. Pedometer readings revealed no significant difference in the intensity of physical activity or duration between the 2 groups.

## Discussion

In overweight or obese patients with type 2 diabetes, a short-term (21 days) nutritional intervention trial using 2 different diets showed that both diets resulted in beneficial effects on metabolic parameters. The macrobiotic Ma-Pi 2 diet was associated with a greater reduction in fasting and post prandial plasma glucose, HbA_1c_, serum cholesterol, HOMA-IR, BMI, and waist and hip circumferences than the standard control diet.

Our study is the first randomized trial to assess and quantify the reported beneficial effects of the Ma-Pi 2 diet versus the standard nutritional recommendations for type 2 diabetes [25]. In this trial patients consuming the Ma-Pi 2 diet experienced a statistically significantly greater benefit in terms of reduced FBG, PPBG, HbA_1c,_ and HOMA–IR, compared to patients receiving the control diet, suggesting that the Ma-Pi 2 macrobiotic diet is a more effective dietary intervention than the standard recommended diet for improving metabolic control in patients with type 2 diabetes. Also, significantly greater weight loss was obtained in the Ma-Pi 2 diet group compared with the control group, despite consumption of the same energy content in both diets. The Ma-Pi 2 diet was also higher in fiber content by up to 10 g/1000 kcal (50%) than the control diet which may also have contributed to the greater weight loss in the Ma-Pi 2 diet group; Langlois et al. conducted a retrospective cohort study of the Canadian population and found that dietary fiber intake was inversely related with incidence of obesity [32].

A reduction in total cholesterol, LDLc and LDL/HDL ratio was observed with both diets, but was significantly higher with the Ma-Pi 2 diet. This could be the result of a higher intake of wholegrain cereals; this is in line with a Cochrane review on the effect of wholegrain cereals on coronary heart disease that found that short-term dietary oatmeal intervention was associated with lower total cholesterol and LDLc [33].

The success of the control diet in improving metabolic control is supported by previous studies on Mediterranean diets. A systematic review and meta-analysis found significant improvement in glycemic control in low-carbohydrate, Mediterranean, and high-protein diets and a greater weight loss in low-carbohydrate and Mediterranean diets compared with their respective control diets [34]; A short trial on the effect of a high-protein/low-carbohydrate diet on glucose control showed a reduction in circulating glucose concentration in patients with untreated type 2 diabetes [35]; A Mediterranean diet, rich in monounsaturated fatty acids and in complex carbohydrate but not high in protein was associated with lower HbA_1c_ levels and 2 hour post-meal glucose levels independently of variations in age, adiposity, energy intake, and physical activity in 901 patients with type 2 diabetes [36].

Overall the positive results obtained with the two diets in our short-term study may be explained in part by the wellbeing (emotional and physical) state of our patients since they were located close to the sea, had a strict control on the caloric intake, perform regular (monitored) physical activity improving their overall quality of life. The greater effect of the Ma-Pi 2 diet compared to the control diet on a number of metabolic parameters may be due to several factors, from changes in inflammation and/or oxidative stress [37] to the composition of microbiota [22]. Ongoing studies should elucidate all these issues, hence a long-term sustainability of the Ma-Pi 2 diet, in particular the acceptance and adherence of the patient to the diet, the implying costs in the patient management remain to be proven.

Patient compliance and adherence to recommended therapeutic diets for diabetes are essential for the diets to be able to produce clinically-significant improvements in patient outcomes, and positive results achieved with diets in clinical trials are often difficult to replicate in real-life practice [38]. In the study presented here, participants attended 2-hour meetings daily for nutritional education and cooking instructions in their respective hotels conducted by a physician and a registered dietitian and/or a cooking instructor. This was done to encourage continuation of the respective diets once the trial was completed.

Our study had a number of limitations including short duration, lack of blinding, and relatively small sample size. The short duration was due to the difficulty in accommodating participants for 24 hours per day and requiring them not to leave their respective hotels for the duration of the trial. A longer-term trial of this type is unlikely to be successful due to the lower number of volunteers and higher number of dropouts that would be likely with increased duration. Participant blinding was impossible due to the distinct differences in the ingredients and therefore taste and appearance of the 2 diets used. Similarly, it was also not possible to blind the medical staff present at the hotels. However, the investigators involved in follow-up and blood testing were blinded to the treatment groups.

## Conclusions

All patients in the Ma-Pi 2 diet group had their glucose levels reduced to the point of being comparable to subjects without type 2 diabetes (target values), following 21-day intervention in a supervised environment. Further long-term follow-up studies are needed to confirm these results and the use of this diet in real-life practice must also be investigated to demonstrate patient acceptance and compliance.

### Availability of supporting data

The data sets supporting the results of this article are included within the article and its additional files.

## Consent

Written informed consent was obtained from the patient for the publication of this report and any accompanying images.

## Abbreviations

(FBG): Fasting blood glucose; (PPBG): Post-prandial blood glucose; (HbA1c): Glycated hemoglobin; (IR): Insulin resistance; (BMI): Body mass index; (95% CI): 95% confidence intervals; (LDLc): Low-density lipoprotein cholesterol; (HDLc): High-density lipoprotein cholesterol; (LDL/HDL ratio): Low-density lipoprotein cholesterol/ High-density lipoprotein cholesterol ratio; (IDF): International Diabetes Federation; (ADA): American Diabetes Association; (OADs): Oral hypoglycemic drugs; (INRAN): National Institute for Food and Nutrition Research; (T0): Baseline; (T21): The 21^st^ day of treatment; (HOMA-IR): Homeostatic model assessment; (AEs): Reported adverse events; (SD): Standard deviation; (ITT) analysis: An intention-to-treat; (DPP-4): Dipeptidyl peptidase-4 inhibitor.

## Competing interests

The authors declare that they have no competing interests.

## Authors’ contributions

AS conducted the study; recruited subjects; collected data; and wrote the manuscript. RDT recruited subjects, conducted the study, and collected data. YMK contributed to study design and to the writing of the manuscript. ER contributed to patients follow-up and reviewed the manuscript. LF recruited subjects, contributed to patients follow-up and reviewed the manuscript. SF analyzed data and contributed to the writing of the manuscript. SA analyzed blood samples and reviewed the manuscript. VF: collected data, and contributed to the writing of the manuscript. FC collected data, and contributed to the writing of the manuscript. VR collected data and contributed to the writing of the manuscript. CP conducted the study, collected data and reviewed the manuscript. ES and RG analyzed data and contributed to the writing and reviewed the manuscript. SM and FF contributed to the study design, to the discussion and reviewed the manuscript. MP conceived and contributed to the study design, and reviewed the manuscript. PP conceived and designed the study, contributed to the discussion and reviewed and edited the manuscript. PP is the guarantor of this work and, as such, had full access to all the data in the study and takes responsibility for the integrity of the data and the accuracy of the data analysis. All authors read and approved the final manuscript.

## Supplementary Material

Additional file 1 MA-PI 2 Macrobiotic Diet Daily Meal Plans and Recipes. Click here for file
