# Supplementary material for: The effect of the macrobiotic Ma-Pi 2 diet vs. the recommended diet in the management of type 2 diabetes: the randomized controlled MADIAB trial
Source: Nutr Metab (Lond). 2014 Aug 25;11:39. doi: 10.1186/1743-7075-11-39 (PMC4190933; doi:10.1186/1743-7075-11-39)
Supplement: Additional file 1 — MA-PI 2 Macrobiotic Diet Daily Meal Plans and Recipes. [file 1743-7075-11-39-S1.doc]

**MA-PI 2 Macrobiotic Diet**

**Daily Meal Plans and Recipes**

**Introductory remarks**

All of the ingredients used to prepare the MA-PI 2 diet recipes (mainly whole grains, vegetables and legumes) were grown, stored and processed without the use of synthetic chemicals. The crops are from old seed varieties, which were produced using natural methods.

All of the products used were labeled, giving exhaustive information on the origin and characteristics of the product and its supply chain (the Pianesian Transparent Label).

No frozen products were used.

Only seasonal vegetables grown in open fields were used. They were fresh, and used within 24 hours of harvesting.

The wandadou jiangyou and miso condiments were non-GMO soy-based fermented foods prepared with the traditional 3-year fermentation process.

Beicha Tea (roasted green tea) was the only drink allowed. The daily intake was about 2 liters per person.

Only unrefined sea salt was used in the various recipes. The average daily intake was 4 g per person.

All of the ingredients were washed accurately; they were rinsed with cool water before being consumed.

Whole grain cereals and legumes were used. Vegetables were either used whole or cut into large pieces.

Only glass and stainless steel cooking tools (knives, ladles, etc.) were used to prepare meals; natural mineral water was also used in the preparation and cooking of meals.

All of the foods and ingredients used in the various recipes were cooked, except for parsley and lettuce. Sesame was lightly toasted before being used in various dishes.

Whole grains and legumes were cooked in pressure cookers.

Whole grain cereals were simply boiled in water with unrefined sea salt; the vegetables used as a condiment for cereals in the various recipes were either cooked together with the cereals or boiled separately and subsequently mixed in with the cooked cereals.

Legumes were boiled in water with Kunbu seaweed (*Laminaria japonica, Aresch*). Unrefined sea salt and other seasoning was added at the end of the cooking time. Chickpeas, azuki and black beans were soaked in mineral water for 12 hours before cooking.

Vegetables were cooked in boiling mineral water, without salt, for just a few minutes to better preserve color and nutrients.

Every meal was immediately served after cooking, especially vegetables, which were served only a few minutes after cooking.

Due to the occasional lack of certain vegetables in the local area, other varieties that were not included in the original Ma-Pi 2 diet, such as broccoli, lettuce, leeks and turnip greens, were used in certain meals during the trial.

**LEGEND:**

**The ingredients for each recipe are displayed in brackets next to the recipe name. Quantities are for a single serving.**

**MA-PI 2 Diet 1900 kcal/day**

**Meal Plan Samples**

**DAY 1 (1900 kcal/day)**

**BREAKFAST**

- Baked millet balls (85 g millet, 8 g sesame, unrefined sea salt)
- Beicha Tea

**SNACK**

- Millet cake (45 g millet, 6 g sesame)
- Beicha Tea

**LUNCH**

- Vegetable soup with barley (20 g barley, 15 g onions, 20 g carrots, 30 g Savoy cabbage, 0.5 g Qundaicai seaweed (*Undaria pinnatifida, Harv*), 1.5 g miso (soy paste))
- Brown rice salad (80 g brown rice, 30 g onions, 10 g broccoli, 2 g wandadou jiangyou (soy sauce), unrefined sea salt)
- Boiled chicory and carrots (200 g chicory, 50 g carrots, 3 g wandadou jiangyou (soy sauce))
- Lettuce with sesame (100 g lettuce, 8 g sesame, unrefined sea salt)
- Lentils (20 g lentils, 0.2 g Kunbu seaweed (*Laminaria japonica, Aresch*), 0.5 g miso (soy paste), unrefined sea salt)
- Beicha Tea

**SNACK**

- Barley croquettes (45 g barley, 5 g parsley, 5 g Haitai seaweed (*Porphyria tenera, Kjell*), 6 g sesame, unrefined sea salt)

**DINNER**

- Vegetable soup (15 g onions, 10 g carrots, 15 g leeks, 20 g chicory, 0.5 g Qundaicai seaweed (*Undaria pinnatifida, Harv*), 3 g wandadou jiangyou (soy sauce))
- Millet with vegetables (95 g millet, 10 g onions, 15 g carrots, 30 g chicory, 30 g cooked lettuce, 2 g wandadou jiangyou (soy sauce), 8 g sesame, unrefined sea salt)
- Chickpeas (20 g chickpeas, 0.2 g Kunbu seaweed (*Laminaria japonica, Aresch*), 0.5 g miso (soy paste), unrefined sea salt)
- Boiled broccoli and chicory (70 g broccoli, 100 g chicory, 2 g yanzimei (pickled ume plums))
- Onion and carrot nituké (40 g onions, 75 g carrots)
- Beicha Tea

**DAY 2 (1900 kcal/day)**

**BREAKFAST**

- Baked millet and brown rice cake with onions (35 g millet, 45 g brown rice, 20 g onions, 20 g carrots, 8 g sesame, 2 g miso (soy paste), unrefined sea salt)
- Beicha Tea

**SNACK**

- Brown rice balls (45 g brown rice, 3 g yanzimei (pickled ume plums), 6 g sesame, unrefined sea salt)
- Beicha Tea

**LUNCH**

- Vegetable and millet soup (15 g millet, 15 g onions, 15 g carrots, 25 g head cabbage, 0.5 g Qundaicai seaweed (*Undaria pinnatifida, Harv*) , 2 g miso (soy paste))
- Brown rice salad (50 g brown rice, 20 g red radishes, 50 g carrots, 2 g wandadou jiangyou (soy sauce), unrefined sea salt)
- Barley with chicory (50 g barley, 15 g onions, 40 g chicory, 1 g miso (soy paste), 10 g sesame, unrefined sea salt)
- Boiled head cabbage with parsley (75 g head cabbage, 4 g parsley)
- Boiled chicory with soy sauce (160 g chicory, 2 g wandadou jiangyou (soy sauce))
- Azuki beans (20 g azuki beans, 0.2 g Kunbu seaweed (*Laminaria japonica, Aresch*), unrefined sea salt)
- Lettuce salad (80 g lettuce, 2 g yanzimei (pickled ume plums))
- Beicha Tea

**SNACK**

- Round millet cakes (45 g millet, 6 g sesame, 2 g miso (soy paste), unrefined sea salt)
- Beicha Tea

**DINNER**

- Vegetable and barley soup (15 g barley, 10 g onions, 10 g carrots, 25 g chicory, 4 g raw parsley, 2 g miso (soy paste))
- Brown rice with carrots and leeks (50 g brown rice, 20 g carrots, 10 g leeks, 8 g sesame, unrefined sea salt)
- Millet salad (50 g millet, 50 g broccoli, unrefined sea salt)
- Chickpeas (20 g chickpeas, 0.2 g Kunbu seaweed (*Laminaria japonica, Aresch*), unrefined sea salt)
- Pressure-cooked broccoli and chicory (50 g broccoli, 175 g chicory)
- Lettuce salad (80 g lettuce, 1 g wandadou jiangyou (soy sauce))
- Beicha Tea

**MA-PI 2 Diet 1700 kcal/day Meal Plan Samples**

**DAY 1 (1700 kcal/day)**

**BREAKFAST**

- Baked millet balls (75 g millet, 8 g sesame, unrefined sea salt)
- Beicha Tea

**SNACK**

- Millet cake (40 g millet, 5 g sesame)
- Beicha Tea

**LUNCH**

- Vegetable soup with barley (15 g barley, 10 g onions, 10 g carrots, 20 g Savoy cabbage, 0.5 g Qundaicai seaweed (*Undaria pinnatifida, Harv*) , 1.5 g miso (soy paste))
- Brown rice salad (80 g brown rice, 15 g onions, 10 g broccoli, 2 g wandadou jiangyou (soy sauce), unrefined sea salt)
- Boiled chicory and carrots (200 g chicory, 40 g carrots, 3 g wandadou jiangyou (soy sauce))
- Lettuce with sesame (100 g lettuce, 8 g sesame, unrefined sea salt)
- Lentils (15 g lentils, 0.2 g Kunbu seaweed (*Laminaria japonica, Aresch*), 0.5 g miso (soy paste), unrefined sea salt)
- Beicha Tea

**SNACK**

- Barley croquettes (40 g barley, 5 g parsley, 5 g Haitai seaweed (*Porphyria tenera, Kjell*), 5 g sesame, unrefined sea salt)

**DINNER**

- Vegetable soup (15 g onions, 10 g carrots, 10 g leeks, 20 g chicory, 0.5 g Qundaicai seaweed (*Undaria pinnatifida, Harv*), 3 g wandadou jiangyou (soy sauce))
- Millet with vegetables (90 g millet, 10 g onions, 15 g carrots, 20 g chicory, 30 g cooked lettuce, 2 g wandadou jiangyou (soy sauce), 8 g sesame, unrefined sea salt)
- Chickpeas (20 g chickpeas, 0.2 g Kunbu seaweed (*Laminaria japonica, Aresch*), 0.5 g miso (soy paste), unrefined sea salt)
- Boiled broccoli and chicory (60 g broccoli, 90 g chicory, 2 g yanzimei (pickled ume plums))
- Onion and carrot nituké (25 g onions, 55 g carrots)
- Beicha Tea

**DAY 2 (1700 kcal/day)**

**BREAKFAST**

- Baked millet and brown rice cake with onions (30 g millet, 40 g brown rice, 17 g onions, 17 g carrots, 8 g sesame, 2 g miso (soy paste), unrefined sea salt)
- Beicha Tea

SNACK

- Brown rice balls (40 g brown rice, 3 g yanzimei (pickled ume plums), 5 g sesame, unrefined sea salt)
- Beicha Tea

LUNCH

- Vegetable and millet soup (15 g millet, 10 g onions, 10 g carrots, 25 g head cabbage, 0.5 g Qundaicai seaweed (*Undaria pinnatifida, Harv*), 2 g miso (soy paste))
- Brown rice salad (45 g brown rice, 20 g red radishes, 50 g carrots, 2 g wandadou jiangyou (soy sauce), unrefined sea salt)
- Barley with chicory (45 g barley, 10 g onions, 30 g chicory, 1 g miso (soy paste), 10 g sesame, unrefined sea salt)
- Boiled head cabbage with parsley (75 g head cabbage, 4 g parsley)
- Boiled chicory with soy sauce (150 g chicory, 2 g wandadou jiangyou (soy sauce))
- Azuki beans (20 g azuki beans, 0.2 g Kunbu seaweed (*Laminaria japonica, Aresch*), unrefined sea salt)
- Lettuce salad (80 g lettuce, 2 g yanzimei (pickled ume plums))
- Beicha Tea

**SNACK**

- Round millet cakes (40 g millet, 5 g sesame, 2 g miso (soy paste), unrefined sea salt)
- Beicha Tea

**DINNER**

- Vegetable and barley soup (10 g barley, 10 g onions, 10 g carrots, 20 g chicory, 4 g raw parsley, 2 g miso (soy paste))
- Brown rice with carrots and leeks (42 g brown rice, 10 g carrots, 10 g leeks, 8 g sesame, unrefined sea salt)
- Millet salad (42 g millet, 40 g broccoli, unrefined sea salt)
- Chickpeas (20 g chickpeas, 0.2 g Kunbu seaweed (*Laminaria japonica, Aresch*), unrefined sea salt)
- Pressure-cooked broccoli and chicory (40 g broccoli, 160 g chicory)
- Lettuce salad (80 g lettuce, 1 g wandadou jiangyou (soy sauce))
- Beicha Tea

**Control Diet**

**Meal Plans and Recipes**

**Introductory remarks**

All of the ingredients used to prepare the individual recipes of the control diet were those commonly available at the market.

Most vegetables were bought at the beginning of the project and stored in a cold room.

Fresh fish was normally used. Seabass and seabream came from fish farms.

The only drink served was natural water. The amount was about two liters per person per day.

The salt used in the various recipes was common table salt. The average daily intake was 4 g per person.

Vegetables were washed and soaked in water for 15 to 20 minutes before use.

The tools used for cooking were made of stainless steel, iron, plastic and glass.

Cooking was done in stainless steel, iron, copper or non-stick cookware, using drinking tap water.

All of the foods used in the various recipes were cooked, except for salads.

Pasta and rice were cooked in a pot of salted boiling water. Condiments were added and mixed in after cooking.

Leafy greens were cooked in salted water for 15 to 25 minutes.

Fish, meat and certain types of vegetables (eggplant, potatoes, peppers, asparagus, artichokes) were usually steamed, grilled, roasted, baked, baked in foil or boiled in salted water.

At every meal, pasta and rice were served immediately after cooking. All soups, meat, fish and cooked vegetables were served 1 to 2 hours on average after cooking.

**LEGEND: The ingredients for each recipe are displayed in brackets next to the recipe name. Quantities are for a single serving.**

**Control diet 1900 kcal/day Meal Plan Samples**

**DAY 1 (1900 kcal/day)**

**BREAKFAST**

- 200 g semi-skimmed cow milk
- 40 g whole wheat crispbreads

**SNACK**

- 200 g apple

**LUNCH**

- Pasta with tomato sauce (100 g durum wheat pasta, 167 g ripe tomatoes, 10 g extra virgin olive oil, 12 g onions, 8 g carrots, 5 g celery, 1 g basil, 10 g grated Parmesan cheese, salt)
- Boiled chard (200 g chard, 5 g extra virgin olive oil, salt)
- Grilled veal fillet (100 g veal fillet, 5 g extra virgin olive oil, salt)
- 30 g whole wheat bread

**SNACK**

- 200 g orange

**DINNER**

- 100 g whole wheat bread
- Sole meunière (100 g sole, 10 g butter, 8 g wheat flour, 5 g lemon juice, 5 g extra virgin olive oil, salt)
- Mixed salad (42 g radicchio, 21 g endive, 21 g lettuce, 42 g fennel, 21 g radish, 42 g orange, 12 g extra virgin olive oil, salt)
- Tomato salad (100 g tomatoes, 5 g extra virgin olive oil, salt)

**DAY 2 (1900 kcal/day)**

**BREAKFAST**

- 200 g semi-skimmed cow milk
- 50 g whole wheat bread

**SNACK**

- 200 g pear

**LUNCH**

- Asparagus with lemon juice (180 g greenhouse asparagus, 11 g extra virgin olive oil, 9 g lemon juice, salt)
- Borlotti beans (70 g dried borlotti beans, 10 g extra virgin olive oil, salt)
- Durum wheat pasta (60 g durum wheat pasta, 10 g extra virgin olive oil, salt)

**SNACK**

- 200 g pineapple

**DINNER**

- Vegetable soup (43 g dried borlotti beans, 21 g carrots, 14 g celery, 36 g potatoes, 29 g zucchini, 14 g fresh peas, 29 g fresh green beans, 11 g extra virgin olive oil, 1 g garlic, 1 g basil, 1 g parsley, salt)
- 100 g whole wheat bread
- Agretti (200 g agretti, 15 g extra virgin olive oil, salt)
- Baked cod (140 g cod, 9 g lemon, 5 g extra virgin olive oil, salt)

**Control diet 1700 kcal/day Meal Plan Samples**

**DAY 1 (1700 kcal/day)**

**BREAKFAST**

- 200 g semi-skimmed cow milk
- 40 g whole wheat crispbreads

**SNACK**

- 200 g apple

**LUNCH**

- Pasta with tomato sauce (100 g durum wheat pasta, 167 g ripe tomatoes, 10 g extra virgin olive oil, 12 g onions, 8 g carrots, 5 g celery, 1 g basil, 10 g grated Parmesan cheese, salt)
- Boiled chard (200 g chard, 3 g extra virgin olive oil, salt)
- Grilled veal fillet (100 g veal fillet, 2 g extra virgin olive oil, salt)
- 30 g whole wheat bread

**SNACK**

- 200 g orange

**DINNER**

- 60 g whole wheat bread
- Sole meunière (100 g sole, 10 g butter, 8 g wheat flour, 5 g lemon juice, 2 g extra virgin olive oil, salt)
- Mixed salad (42 g radicchio, 21 g endive, 21 g lettuce, 42 g fennel, 21 g radish, 42 g orange, 12 g extra virgin olive oil, salt)
- Tomato salad (100 g tomatoes, 3 g extra virgin olive oil, salt)

**DAY 2 (1700 kcal/day)**

**BREAKFAST**

- 200 g semi-skimmed cow milk
- 50 g whole wheat bread

**SNACK**

- 200 g pear

**LUNCH**

- Asparagus with lemon juice (180 g greenhouse asparagus, 11 g extra virgin olive oil, 9 g lemon juice, salt)
- Borlotti beans (60 g dried borlotti beans, 5 g extra virgin olive oil, salt)
- Durum wheat pasta (60 g durum wheat pasta, 5 g extra virgin olive oil, salt)

**SNACK**

- 200 g pineapple

**DINNER**

- Vegetable soup (43 g dried borlotti beans, 21 g carrots, 14 g celery, 36 g potatoes, 29 g zucchini, 14 g fresh peas, 29 g fresh green beans, 11 g extra virgin olive oil, 1 g garlic, 1 g basil, 1 g parsley, salt)
- 80 g whole wheat bread
- Agretti (200 g agretti, 10 g extra virgin olive oil, salt)
- Baked cod (140 g cod, 9 g lemons, 5 g extra virgin olive oil, salt)
